# Characterization of the Alkaline Laccase Ssl1 from *Streptomyces sviceus* with Unusual Properties Discovered by Genome Mining

**DOI:** 10.1371/journal.pone.0052360

**Published:** 2012-12-20

**Authors:** Matthias Gunne, Vlada B. Urlacher

**Affiliations:** 1 Institute for Biochemistry, Heinrich-Heine-Universität Düsseldorf, Düsseldorf, Germany; Institute of Molecular Genetics IMG-CNR, Italy

## Abstract

Fungal laccases are well investigated enzymes with high potential in diverse applications like bleaching of waste waters and textiles, cellulose delignification, and organic synthesis. However, they are limited to acidic reaction conditions and require eukaryotic expression systems. This raises a demand for novel laccases without these constraints. We have taken advantage of the laccase engineering database LccED derived from genome mining to identify and clone the laccase Ssl1 from *Streptomyces sviceus* which can circumvent the limitations of fungal laccases. Ssl1 belongs to the family of small laccases that contains only few characterized enzymes. After removal of the twin-arginine signal peptide Ssl1 was readily expressed in *E. coli.* Ssl1 is a small laccase with 32.5 kDa, consists of only two cupredoxin-like domains, and forms trimers in solution. Ssl1 oxidizes 2,2′-azino-bis(3-ethylbenzthiazoline-6-sulfonic acid) (ABTS) and phenolic substrates like 2,6-dimethoxy phenol, guaiacol, and syringaldazine. The k_cat_ value for ABTS oxidation was at least 20 times higher than for other substrates. The optimal pH for oxidation reactions is substrate dependent: for phenolic substrates the highest activities were detected at alkaline conditions (pH 9.0 for 2,6-dimethoxy phenol and guaiacol and pH 8.0 for syringaldazine), while the highest reaction rates with ABTS were observed at pH 4.0. Though originating from a mesophilic organism, Ssl demonstrates remarkable stability at elevated temperatures (T_1/2,60°C_ = 88 min) and in a wide pH range (pH 5.0 to 11.0). Notably, the enzyme retained 80% residual activity after 5 days of incubation at pH 11. Detergents and organic co-solvents do not affect Ssl1 stability. The described robustness makes Ssl1 a potential candidate for industrial applications, preferably in processes that require alkaline reaction conditions.

## Introduction

Although significant progress has been achieved in enzyme engineering, the discovery and characterization of novel enzymes from diverse (micro)organisms still plays an essential role for the development of biocatalytic processes. Especially in the case of laccases it has been demonstrated that very few positions can be mutated without loss of activity [1]. This is due to highly conserved functionally essential regions of these enzymes. Laccases (EC 1.10.3.2, *p*-diphenol:dioxygen oxidoreductase) belong to the enzyme family of multicopper oxidases. They catalyze the one-electron oxidation of substrate molecules and transfer thereby abstracted electrons to molecular oxygen which is reduced to water. The electrons are channeled through highly conserved copper binding residues from the substrate oxidizing T1 copper site to the T2/T3 trinuclear copper cluster where oxygen is bound and reduced to water by four electrons [2]. Typical substrates are mono-, di- and polyphenols, hydroxylated aryls, aromatic or aliphatic amines and metal ions. Most laccases show low substrate specificity and accept a large variety of different substrates. It was proposed that the redox potential at the T1 site is the key factor determining whether a substrate can be oxidized by laccase [3].

The most exhaustively investigated laccases originate from white-rot fungi such as *Trametes* sp. or *Pleurotus* sp. Many of those fungal laccases exhibit high redox potentials and therefore possess high activities towards their substrates. However, owing to pH preference and stability [4], their use is restricted to acidic reaction conditions and mesophilic temperatures. Moreover, fungal laccases are highly glycosylated enzymes and cannot be produced with bacterial expression systems. Recent approaches based on metagenomic libraries [5] or available and fast growing sequence data [6] demonstrate the wide distribution of laccases or laccase-like enzymes in bacteria. Sirim et al. classified more than 2200 laccases and related enzymes from available genome sequences and structural data and assigned more than 1000 potential bacterial laccases into 5 different superfamilies [7].

The physiological functions of most characterized bacterial laccases remain unknown. The few described functions include spore pigmentation as found for the laccase CotA from *B. subtilis* [8] and copper homeostasis as suggested for CueO from *E. coli* [9], [10] and CopO from *Corynebacterium glutamicum* [11].

By now, the characteristics and biotechnological potential of these enzymes are poorly investigated and still few reports on bacterial laccases have been published. Nevertheless, these reports demonstrate the thermal robustness and more alkaline activity profiles of bacterial laccases compared to fungal enzymes. Exemplarily, the laccase from *Thermus thermophilus* shows extreme stability at high temperatures with a half-life of thermal inactivation at 80°C of more than 14 h [12], and laccases from *Bacillus halodurans* and *Streptomyces coelicolor* exhibit maximum activities towards syringaldazine or 2,6-dimethoxyphenol at pH values of 7.5 or 9.4 [13], [14]. This kind of bacterial alkaline laccase may circumvent the limitations of fungal laccases and extend the range of feasible reaction conditions in industrial applications of laccase towards higher pH values, elevated reaction temperatures and prolonged production processes owing to more robust biocatalysts.

Similar to other multicopper oxidases, laccases commonly consist of three cupredoxin-like domains with the T1 copper coordinated by two histidines and a cysteine residue in domain 3 and the trinuclear T2/T3 cluster at the interface of domain 1 and 3 coordinated by eight histidines [15]. In 2002, a novel type of laccase was described which showed low sequence similarity to known eukaryotic and bacterial laccases and a smaller molecular size [16], [17] due to lack of the second domain present in most laccases [14].

Here, we describe the cloning, expression and characterization of the small two-domain Ssl1 laccase from *Streptomyces sviceus*. Ssl1 demonstrated moderate thermostability, alkaline pH-activity profile, and stability in a wide pH range up to pH 11 and in presence of organic solvents. Thereby its catalytic properties were distinct from other laccases.

## Materials and Methods

### Materials and Strains

All reagents were of analytical grade or higher and purchased from commercial sources. Enzymes for molecular cloning, nucleotide ladders and protein ladders were obtained by Fermentas (St. Leon-Rot, Germany). Molecular cloning and plasmid propagation were carried out in *E. coli* DH5α (Novagen, Darmstadt, Germany). *E. coli* BL21(DE3), *E. coli* BL21(DE3) pLys, *E. coli* Rosetta(DE3) (all from Novagen) and *E. coli* BL21-CodonPlus (DE3)-RP (Stratagene, Waldbronn, Germany) served as expression hosts. Genomic DNA of *Streptomyces sviceus* (DMS 924) was purchased from the DSMZ (Braunschweig, Germany).

### Cloning of *ssl1*

The *ssl1* gene (SSEG_02446) was amplified by PCR with the primers CTTgctagcATGCATCATCATCATCATCATGCCCCGGGCGGCGAG and GGCaagcttTCAGTGGTGGTGTTCGGCCCGC (Eurofins MWG Operon, Ebersberg, Germany) using genomic DNA of *S. sviceus* as template. NheI and HindIII endonuclease recognition sites are shown in lowercase, the sequence of the hexahistidine tag is underlined. The genomic sequence of *ss1l* was truncated at the 5′ end in order to remove a natural signal sequence of the twin arginine translocation pathway. The PCR product was purified and cloned into the pET22H plasmid [18] using the *Nhe*I and *Hind*III restriction endonucleases. The sequence of the *ssl1* insert in the resulting pET22-ssl1 plasmid was verified by sequencing (Eurofins MWG Operon).

### Expression Optimization and Purification of Ssl1

*E. coli* expression strains were transformed with pET22ssl1 and grown in 200 mL medium containing ampicillin (100 µg ml^−1^) and, when required, chloramphenicol (34 µg ml^−1^). Cultures were grown at 30°C or 37°C and 140 rpm. Expression conditions were optimized with regard to *E. coli* expression strain (BL21(DE3), BL21(DE3) pLys, Rosetta(DE3), BL21-CodonPlus (DE3)-RP), medium (LB, TB, M9, 2xYT), induction OD_600_ (0.5, 1, 1.5, 2), inducer concentration (10 µM, 40 µM, 200 µM, 1 mM), expression temperature (20°C, 25°C, 30°C, 37°C) and expression duration (3 to 24 h).

Highest volumetric activities were obtained in *E. coli* BL21-CodonPlus (DE3)-RP grown at 30°C and 140 rpm in TB medium, when expression was induced at an OD_600_ of 1.0 with 40 µM IPTG. 2 mM copper(II) sulfate were added to the culture medium upon induction, and the expression was carried out for 8 h at 25°C and 140 rpm.

After expression, cultures were harvested by centrifugation at 11000 g at 4°C for 20 min. The cell pellets were resuspended in potassium phosphate buffer (50 mM, pH 7.5) containing 0.1 mM PMSF and 0.3 mM copper(II) sulfate. Cell disruption was done by sonication with a Branson sonifier SLPe (3 cycles: energy pulse mode for 90 s at 50% amplitude with 10 J, 2 s off time) with at least 1 min on ice between the cycles. Cell debris was removed by centrifugation at 31000 g for 30 min at 4°C. The soluble fraction was incubated for 20 min at 65°C and precipitate was removed by centrifugation at 48000 g for 30 min at 4°C. The resulting supernatant was filtered through a cellulose acetate membrane with 0.45 µm pores. For purification of Ssl1 by immobilized metal affinity chromatography (IMAC), the filtrate was loaded onto a Talon (BD Biosciences, Heidelberg, Germany) packed and preequilibrated gravity flow column (7 mL bed volume). The column was washed with 5 column volumes (CV) equilibration buffer (50 mM potassium phosphate, pH 7.5, 500 mM sodium chloride) and,thereafter, with 5 CV washing buffer (50 mM potassium phosphate, pH 7.5, 500 mM sodium chloride, 5 mM imidazole). Ssl1 was eluted in 1 CV elution buffer (50 mM potassium phosphate, pH 7.5, 500 mM sodium chloride, 100 mM imidazole). The eluate was concentrated with Vivaspin 15 columns (10 kDa cut-off, Sartorius, Göttingen, Germany) and imidazole and sodium chloride were removed by use of PD miditrap G-25 desalting columns (GE Healthcare, München, Germany). Purified Ssl1 was stored at -20°C without loss of activity until use. The purity of Ssl1 was estimated by sodium dodecyl sulfate-polyacrylamide gel electrophoresis (SDS-PAGE) on a 12.5% gel.

### Spectroscopy and Light Scattering

The absorption spectrum of Ssl1 was determined in the range of 300 to 700 nm with a Lamdba 35 spectrophotometer (Perkin Elmer, Rodgau, Germany). The solution contained 125 µM Ssl1 in 50 mM MOPS buffer at pH 7.5. Ssl1 concentrations were determined by the Bradford method with BSA as standard, calculation of molar concentrations of Ssl1 were based on the computationally determined and experimentally verified molar mass of 32.5 kDa.

The copper content of Ssl1 was determined by atom absorption spectroscopy with an AAnalyst 100 (Perkin Elmer, Rodgau, Germany) at 324.8 nm with a slit width of 0.7 nm.

The molar mass of Ssl1 in native form was determined by multi-angle static light scattering. Therefore, Ssl1 was applied to an equilibrated Superdex 200 10/300 GL size exclusion chromatography (SEC) column mounted on an ÄKTA purifier FPLC system (GE Healthcare, München, Germany). The SEC was conducted at 0.5 mL min^−1^ flow with 50 mM potassium phosphate buffer at pH 7.5. The injection volume was 100 µL with a Ssl1 concentration of 7.5 mg mL^−1^. Refraction index and scattering intensity were measured on-line by an Optilab rEX differential refractometer and a miniDAWN TREOS triple-angle light scattering detector (both Wyatt Technology Europe, Dernbach, Germany). Data were integrated and analyzed by the Astra software package.

### Activity Assays

The activity of Ssl1 towards the substrates 2,6-dimethoxyphenol (2,6-DMP), 2,2′-azino-bis(3-ethylbenzothiazoline-6-sulphonic acid (ABTS), guaiacol, and syringaldazine (SGZ) was tested in 50 mM McIlvaine’s buffer (pH 3–6), 50 mM potassium phosphate buffer (pH 7–8), 50 mM glycine-sodium hydroxide buffer (pH 9–10) and 50 mM 3-(cyclohexylamino)-1-propanesulfonic acid (CAPS)-sodium hydroxide buffer (pH 11) at room temperature. The oxidation of 2,6-DMP was followed spectrophotometrically at 468 nm (ε = 49.6 mM^−1^ cm^−1^), the reaction mixture contained 0.5 mM 2,6-DMP. The oxidation of ABTS (0.5 mM) was followed at 420 nm (ε = 36 mM^−1^ cm^−1^), oxidation of guaiacol (2 mM) to the dimeric product tetraguaiacol was followed at 470 nm (ε = 26 mM^−1^ cm^−1^) and oxidation of SGZ (50 µM, 10% dimethyl sulfoxide) was followed at 525 nm (ε = 65 mM^−1^ cm^−1^). Ssl1 activity towards tyrosine was tested at pH 4.0, 7.0 and 9.0. The reaction contained 0.1 mM tyrosine and 2 µM Ssl1, oxidation was monitored at 280 nm (ε = 4.4 mM^−1^ cm^−1^). Activity assays were performed in triplicate. For pH activity profiles, activities at optimal pH were set as 100%.

For testing thermal stability, Ssl1 (75 µM in 50 mM glycine-sodium hydroxide buffer with pH 9.0) was incubated at 50°C, 60°C, 70°C and 80°C for 0 to 240 min. After incubation, samples were immediately chilled on ice. Residual activities were measured with 2,6-dimethoxyphenol as described above and the half-time of activity loss was determined by non-linear regression to the equation of exponential decay (*a*(*t*) = *a*_0_ exp (-*t* ln2/*T*_1/2_)) by OriginPro 8.5 (OriginLab Corporation, Northampton, MA, USA). Data were collected as triplicate.

For testing the stability at different pH values, Ssl1 (75 µM) was incubated at pH 3, 4, 5, 6 (in 50 mM McIlvaine’s buffer), 7, 8 (in 50 mM potassium phosphate buffer), 9, 10 (in 50 mM glycine-sodium hydroxide buffer) and 11 (in 50 mM CAPS-sodium hydroxide buffer) at room temperature and residual activities were determined with 2,6-dimethoxyphenol.

Relative activities of Ssl1 in presence of organic solvents were determined after 10 min incubation in glycine-sodium hydroxide buffer (50 mM, pH 9.0) containing solvent in indicated concentrations at ambient temperature. In case of water immiscible solvents (isooctane and *n*-hexane), samples were agitated with 600 rpm in a thermomixer for the same period. Activities were measured with 2,6-dimethoxyphenol as described above. Stability towards organic solvents was accessed by incubation with solvent for 20 h at ambient temperature and subsequent activity measurement with 2,6-dimethoxyphenol.

Kinetic constants of Ssl1 were determined with the substrates 2,6-DMP, ABTS and SGZ. Reactions contained 3.5 µM Ssl1 and 0 to 6 mM 2,6-DMP in 50 mM glycine-NaOH buffer (pH 9.0), 2.3 µM Ssl1 and 0 to 10 mM ABTS in 50 mM McIlvaine buffer (pH 4.0), or 22 nM Ssl1, 0 to 40 µM SGZ and 2% dimethyl sulfoxide in 50 mM potassium phosphate buffer (pH 8.0). The reactions were conducted at room temperature and followed spectrophotometrically at 468 nm (2,6-DMP, ε = 49.6 mM^−1^ cm^−1^), 420 nm (ABTS, ε = 36 mM^−1^ cm^−1^) and 525 nm (SGZ, ε = 65 mM^−1^ cm^−1^). Initial rates were determined in triplicate and resulting data were fitted by non-linear regression to the hyperbolic equation (*v* **=** *v_max_* [*S*]/(*K_m_*+[*S*])) in OriginPro 8.5.

## Results and Discussion

### Sequence Analysis and Cloning of ssl1 from *Streptomyces sviceus*

**Figure 1:**
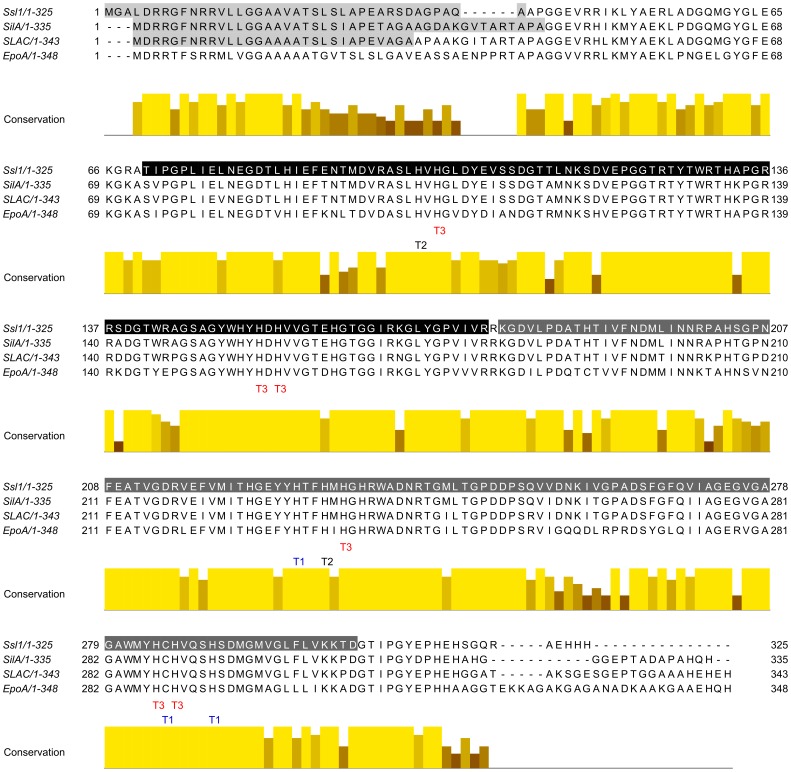
Multiple sequence alignment of homologue small laccases. Ssl1 from *Streptomyces sviceus*, SilA from *S. ipomoea*, EpoA from *S. griseus* and SLAC from *S. coelicolor*. The copper binding residues are conserved in all 4 laccases as indicated. All four laccases consist of 2 domains (indicated in Ssl1, black: domain 1, dark grey: domain 2). Signal sequences of the twin-arginine pathway are indicated in light grey. Sequence identity between the four laccases is high and variations are mainly located at the termini.

*S. sviceus* is a mesophilic soil bacterium best known for its ability to produce the glutamine antagonist acividin (or U-42,126) [19]. According to the Laccase Engineering Database [7] *S. sviceus* contains two laccases of different type, one belonging to SUBfamiliy K of SLAC homologues from *S. coelicolor* and the second belonging to SUBfamily J with homologues of CueO from *E. coli*. Here we describe the cloning, expression and characterization of the laccase Ssl1 (*S. sviceus* laccase 1) from SUBfamiliy K. To this subfamily belong also the previously described EpoA laccase from *Streptomyces griseus*, SLAC laccase from *S. coelicolor*, and SilA laccase from *S. ipomoea*. An alignment of the Ssl1 amino acid sequence with these two-domain laccases (Fig. 1) demonstrates the high sequence similarity within this family. All copper coordinating residues (one cysteine and ten histidins) in these laccases are strictly conserved and conservation among neighboring amino acids is high. Sequence variations are mainly located at both termini, including the tat signal, and in some regions that were found to form loops in the crystal structure of SLAC.

As indicated by a Conserved Domain Search [20] Ssl1 seems to possess an architecture of two cupredoxin-like domains missing domain 2 of large laccases. Analysis of the amino acid sequence by SignalP 4.0 server [21] revealed a potential signal sequence spanning the first 39 amino acids and belonging to the twin-arginine translocation (tat) pathway. In contrast to the sec pathway, translocation via the tat pathway allows secretion of fully folded and cofactor-bound enzymes. A bioinformatics analysis of known laccases showed that 76% of bacterial laccases contain a secretion signal [6]. Cloning and expression of *ssl1* containing the tat signal did not result in active enzyme. Therefore, *ssl1* was amplified without the signal sequence. For simple purification of Ssl1 by immobilized metal affinity chromatography (IMAC) a 6-hexahistidine tag was introduced. The resulting 882 bp PCR fragment was cloned into pET22H [18] to give pET22-ssl1.

### Recombinant Expression and Purification of Ssl1

Highest volumetric activities of Ssl1 were obtained with *E. coli* BL21-CodonPlus (DE3)-RP as expression host (800–1000 U L^−1^). This expression strain contains a plasmid encoding rare tRNAs for arginine and proline that are frequently found in genes of GC-rich genomes such as in *Streptomyces* strains. In case of Ssl1 there are three prolines (P72, P206 and P248) and four arginines (R46, R54, R68 and R200) that are encoded by the CCC or AGG codon which are transcribed by two of the CodonPlus-RP tRNAs. Since *ssl1* contains further codons that are rarely used in *E. coli*, codon optimization of the *ssl1* nucleotide sequence can be expected to increase expression efficiency and volumetric activities.

**Figure 2:**
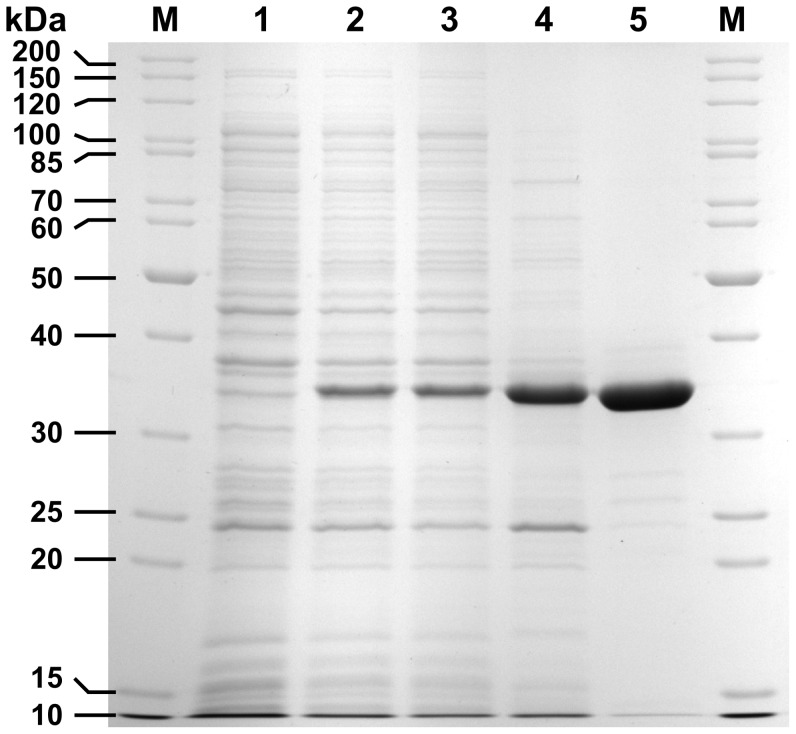
SDS-PAGE analysis of recombinant expression and purification process of Ssl1. Ssl1 migrates at 33 kDa. It was expressed in *E coli-*CodonPlus (DE3)-RP and purified to homogeneity by heat precipitation and immobilized metal affinity chromatography (IMAC). M: molecular size marker, lane 1: cell extract before induction, lane 2: cell extract after expression, lane 3: soluble fraction from cell disruption, lane 4: soluble fraction from heat precipitation, lane 5: IMAC eluate.

For optimal Ssl1 expression, cultures were grown in TB medium at 30°C until OD_600_ reached 1.0 when expression was induced by only 40 µM IPTG with 2 mM copper sulfate as supplement. After 16 h at 25°C the cells were harvested and lysed. The soluble fraction was incubated for 20 min at 65°C which lead to precipitation of most *E. coli* proteins while the activity of Ssl1 remained stable. Precipitated proteins were removed and the lysate was applied to a single IMAC purification step which resulted in homogeneously pure Ssl1 as judged by SDS-PAGE (Fig. 2). Typical expressions yielded 40 to 50 mg Ssl1 per liter of culture with specific activity of 21.7±0.25 U mg^−1^ (with ABTS as substrate).

As determined by atomic absorption spectroscopy Ssl1 contained 2.5 copper ions per molecule. According to the four canonical copper binding sites of laccase, we expected four copper equivalents instead of 2.5. The used expression and purification strategy resulted in partially copper depleted enzyme. Partial copper depletion of bacterial laccases when heterologously expressed in *E. coli* was observed repeatedly [22]–[24]. Since all four canonical copper ions are required for activity copper depletion during expression and purification limits the obtainable yield of active laccase. This might be due to the high level of protein expressed under the control of the strong T7-promoter, which is not completely loaded with copper ion upon expression.

In SDS-PAGE Ssl1 migrated at 33 kDa corresponding to the theoretically determined molecular weight of 32.5 kDa. When incubated in loading buffer containing SDS but without reducing agent, Ssl1 migrated at approximately 100 kDa (not shown). Accordingly, in multiangle static light scattering experiments the molecular weight of active Ssl1 was determined to be 98.3 kDa. This molecular weight corresponds to a homotrimeric oligomerization state of active Ssl1. Other two-domain laccases were found to form either homotrimers (EpoA, [16]) or homodimers (SilA, [25]), for SLAC both forms were observed [14], [26]. The need for oligomerization of two-domain laccases was attributed to the fact that the trinuclear cluster is located at the interface of domain 1 and 2 of neighboring laccase molecules, thereby forming catalytic entities of electronically connected T1 and T2/T3 copper centers as seen in the SLAC crystal structure [26]. In three-domain laccases, domain 2 connects the domains 1 und 3 in a fashion that enables the formation of the trinuclear center at the interface of domain 1 and 3 of a single enzyme molecule. Owing to the lack of domain 2, in two-domain laccases this is not possible. Thus, formation of the trinuclear cluster is achieved by assembly into an appropriate quarternary structure.

### UV-vis Spectrum of Ssl1

**Figure 3:**
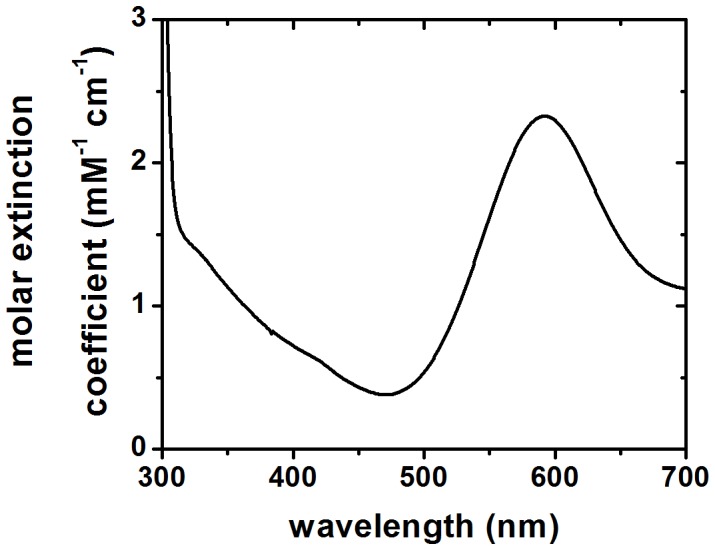
UV/vis spectrum of Ssl1 in potassium phosphate buffer. Ssl1 shows a laccase-typical absorption spectrum. The maximum at 592 nm (ε_Ssl1, 592 nm_ = 2.796±0.191 mM^−1^ cm^−1^) corresponds to type I or blue copper and the shoulder around 330 nm is characteristic for type 3 copper centers.

Purified Ssl1 solutions are characterized by a deep blue color. Accordingly, the absorption spectrum of Ssl1 in the range of 300 to 700 nm (Fig. 3) showed the typical laccase features, with a maximum at 592 nm corresponding to the T1 copper center and a shoulder at approximately 330 nm reflecting the binuclear T3 copper center. The maximum at 592 nm can be utilized for quantification of Ssl1 (ε = 2.796±0.191 mM^−1^ cm^−1^).

### Biochemical Properties of Ssl1

**Figure 4:**
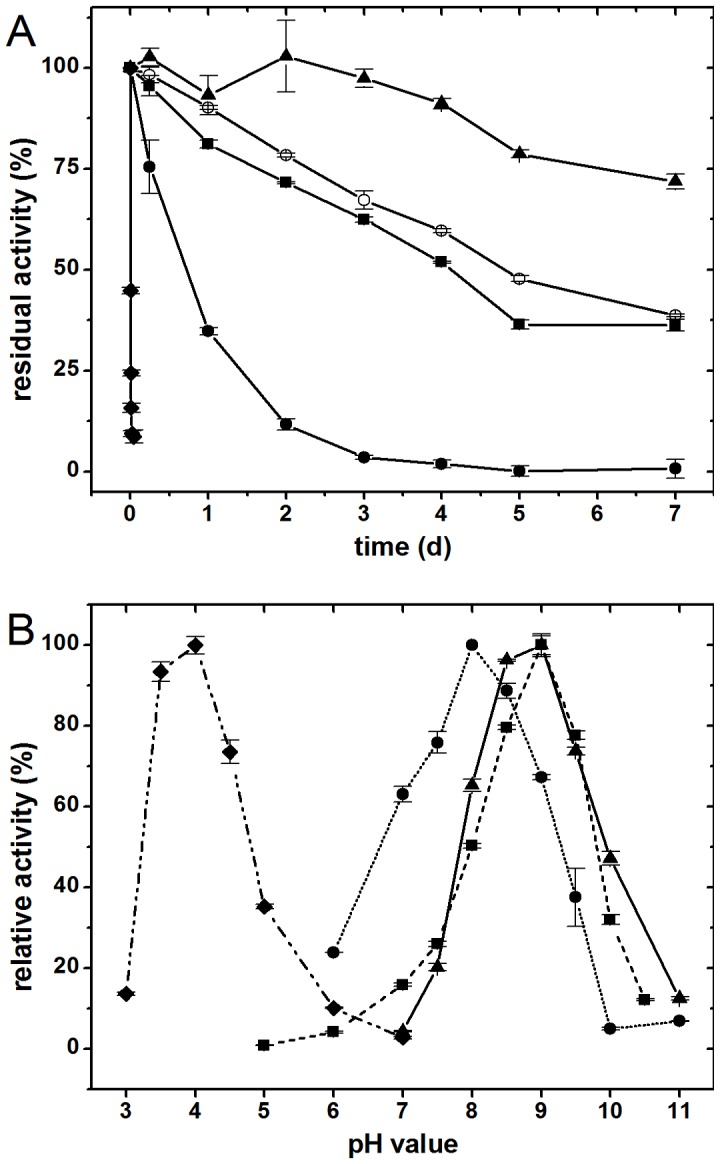
pH optima and pH stability of Ssl1. A: Stability of Ssl1 in buffers with different pH values was tested as residual oxidation activity towards 2,6-dimethoxy phenol. Ssl1 was inactivated within 30 min at pH 3 (values shown as diamonds), and within 3 days at pH 4 (circles). Stability at pH 5 (open circles) and pH 7 (squares) was similar with a half-time around 4 to 5 days. At pH 11 (triangles) Ssl1 was most stable with more than 70% residual activity after 7 days. B: Relative activities of Ssl1 at different pH values towards the substrates ABTS (values shown as diamonds), syringaldazine (circles), 2,6-dimethoxy phenol (triangles) and guaiacol (squares). All activities were normalized to the values at optimum pH with the respective substrate. Optimal pH values are 4 for ABTS, 8 for syringaldazine, and 9 for 2,6-dimethoxy phenol and guaiacol.

Ssl1 was able to oxidize syringaldazine but did not accept tyrosine as substrate. In combination with the typical UV-vis spectrum and conservation of the canonical copper binding residues, Ssl1 could be classified as laccase, not as tyrosinase. Ssl1 oxidized a range of other typical laccase substrates like 2,6-DMP and guaiacol. ABTS was also oxidized by this enzyme. Thereby the activity-pH dependence was bell-shaped for all substrates (Fig. 4). Maximal oxidation activity towards ABTS was reached at pH 4.0 as observed for most laccases with this substrate, whereas Ssl1 activity peaked at alkaline pH values with each of the phenolic substrates 2,6-DMP, guaiacol (both pH 9.0) and syringaldazine (pH 8.0).

The bell-shaped activity profiles with phenolic substrates can be ascribed mainly to two antagonistic effects: (a) the redox potential of phenolic substrates decreases with increasing pH, which results in a larger redox potential difference of substrate and T1 copper and thus in higher activity; (b) hydroxide ions bind at the trinuclear cluster and inhibit the oxygen reduction which reduces activities at high pH [27], [28]. The observed optimum at pH 9 is one of the most alkaline activity optima of laccases reported so far. Generally, fungal laccases are active under acidic conditions [4] and only few bacterial laccases show activity in alkaline milieu [29]. For certain industrial processes like addition of laccase to washing powder, decolourization of waste waters, or treatment of Kraft pulps, where alkaline reaction milieus prevail, alkaline activity would be preferable.

Ssl1 reactions followed Michaelis-Menten kinetics and analysis of kinetic parameters showed higher catalytic efficiency for ABTS (20.6 s^−1^ mM^−1^) than for syringaldazine (3.66 s^−1^ mM^−1^) and 2,6-DMP (0.361 s^−1^ mM^−1^). Observed K_m_ values were in the micromolar range, turn over numbers (k*_cat_*) strongly depended on the substrate and ranged from 443 min^−1^ for ABTS to 19.3 min^−1^ and 3.47 min^−1^ for 2,6-DMP and syringaldazine, respectively (Table 1).

**Table 1:** 

laccase	ABTS			2,6-dimethoxy phenol			syringaldazine			
	pH optimum	*K*_m_ (mM^−1^)	*k*_cat_ (min^−1^)	pH optimum	*K*_m_ (mM^−1^)	*k*_cat_ (min^−1^)	pH optimum	*K*_m_ (µM^−1^)		*k*_cat_ (min^−1^)
Ssl1	4.0	0.36	443	9.0	0.89	19.3	8.0	15.8		3.47
EpoA[16]	n.a.	n.a.	n.a.	not oxidized			not oxidized			
SilA[25]	5.0	0.40	599	8.0	4.27	252	8	n.a.	n.a.	
SLAC[14], [35]	4.0	n.a.	n.a.	9.4	∼4	∼270	n.a.	n.a.	n.a.	

n.a.: not available.

Despite high similarity to other two-domain laccases (Ssl1 shares 84% identity with SLAC, 88% with SilA, and 74% with EpoA), the catalytic features of these four two-domain laccases differ substantially. It is notable that EpoA could not oxidize 2,6-DMP, guaiacol and syringaldazine. SLAC and SilA accept these substrates but show catalytic constants that differ from the constants measured for Ssl1. For 2,6-DMP oxidation by Ssl1 the K_m_ was lower by a factor of 5 and k*_cat_* by a factor of 14 compared to SilA and SLAC. Further, SilA and SLAC show shifted pH optima (see Table 1). In a future comparative study, ideally on structural level, the minor sequence variations in these four enzymes would help to understand the role that the differing residues play in substrate binding and oxidation.

### Stability of Ssl1

Ssl1 showed remarkable stability with regard to pH, temperature and presence of organic solvents. In a broad pH range from 5 to 10, Ssl1 retained 40 to 60% residual activity after 5 days of incubation (Fig. 4). At more acidic conditions it was less stable, at pH 4 Ssl1 lost 65% activity within 1 day and at pH 3 Ssl1 was almost completely inactivated within 30 min. Interestingly, the highest stability was observed at pH 11 with approximately 80% residual activity after 5 days incubation. Stability at high pH values can be explained by the fact that inhibition of the trinuclear cluster by hydroxide ions reduces auto-oxidation of laccase and thereby stabilizes the enzyme [30].

Ssl1 also showed moderate stability at elevated temperatures, the half-times of residual activities were 226±12 min at 50°C, 88±9 min at 60°C, 29±4 min at 70°C and 10±0.4 min at 80°C. This thermal robustness was utilized in the purification process by heat precipitation of most *E. coli* host proteins, while Ssl1 activity remained unaltered. Thermal stability is not only beneficial for this simple and efficient purification step, but is also an ideal prerequisite for directed evolution experiments since stable enzymes can tolerate more destabilizing mutations and thereby allow screening within a larger mutational space as was shown e.g. for P450 monooxygenases [31]. Moreover, thermal stability is usually considered as beneficial for industrial processes since it is often connected to operational stability of the enzyme which allows higher reaction temperature, longer process duration, and in general a more flexible process management.

**Figure 5:**
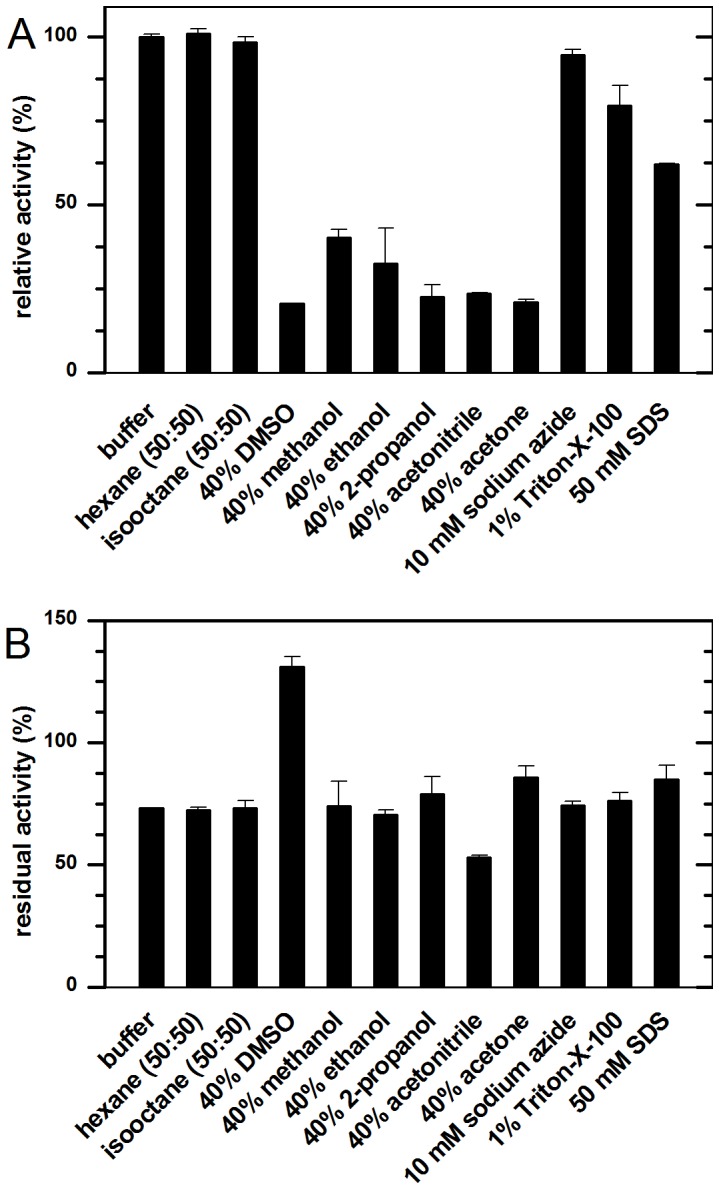
Effect of detergents, salts and organic co-solvents on Ssl1. A: Relative activity of Ssl1 in presence of organic solvents, salts and detergents towards 2,6-dimethoxyphenol oxidation. Water immiscible solvents did not affect Ssl1 activity, with water miscible co-solvents the activity dropped to 20–40%. Ssl1 tolerated the addition of 10 mM sodium azide, with the detergents SDS and Triton-X-100 the activity was reduced to 60 or 80%. B: Residual activity of Ssl1 after 20 h incubation with organic solvents, salts and deteregents. DMSO stabilized Ssl1 whereas acetonitrile lead to a reduction in residual activity. All other studied additives did not lead to major changes of Ssl1 stability.

Further, stability of Ssl1 in the presence of several organic solvents was examined. Presence of a second phase of the water immiscible organic solvents *n*-hexane and isooctane did not alter either activity or stability of the enzyme (Fig. 5). With 40% water miscible solvents like DMSO, methanol, ethanol, 2-propanol, acetonitrile or acetone in the reaction system, the activity dropped to 20 to 40%. However, the stability of Ssl1 remained unchanged with most solvents and about 75% residual activity were detected after 20 h. Ssl1 was destabilized by acetonitrile (53% residual activity), whereas DMSO acted as stabilizer and adaption over 20 h even increased measured activities to 131%. Addition of 50 mM sodium dodecyl sulfate or 1% Triton-X-100 lead to a reduction of activity to 62 and 79% but showed no effect on Ssl1 stability. Addition of 10 mM sodium azide, a well-known laccase inhibitor [32], led to a slight decrease of activity by 5%, whereas many laccases are completely inhibited by concentrations in the micromolar range [33], [34]. The relative stability of Ssl1 with organic co-solvents and other chemicals allows use of the enzyme in a wide variety of reaction compositions. This is particularly useful since many described laccase substrates, like polyaromatic hydrocarbons or phenylpropanoids, are poorly soluble in water and use of an additional organic phase as substrate reservoir in the reaction could facilitate the conversion of higher amounts of substrate. Further the robustness of Ssl1 activity enables its use in processes where reaction compositions cannot be entirely controlled. E.g. waste waters will usually contain a diverse mixture of all kinds of chemical compounds that might interfere with enzyme activity. Since Ssl1 tolerated all studied additives to a certain degree, its use in such undefined reaction compositions is feasible. Since Ssl1 showed both, thermal stability and stability in presence of additives, we conclude that it possesses the operational stability required for biocatalytic processes.

### Conclusions

Ssl1 from *S. sviceus* is a small two-domain laccase with unusual properties. It can be easily expressed in *E. coli* and combines stability in a wide pH range, at elevated temperatures and in presence of organic solvents with an alkaline activity profile. This makes Ssl1 a suitable candidate for industrial biocatalysis, especially in processes that cannot be accessed by other laccases due to the requirement of high pH values or organic co-solvents.
